# Surveys on *Coxiella burnetii* infections in Swedish cattle, sheep, goats and moose

**DOI:** 10.1186/1751-0147-56-39

**Published:** 2014-07-09

**Authors:** Anna Ohlson, Jonas Malmsten, Jenny Frössling, Göran Bölske, Anna Aspán, Anne-Marie Dalin, Ann Lindberg

**Affiliations:** 1 Department of Disease Control and Epidemiology, National Veterinary Institute, SE-751 89 Uppsala, Sweden; 2 Department of Pathology and Wildlife Diseases, National Veterinary Institute, SE-751 89 Uppsala, Sweden; 3 Department of Bacteriology, National Veterinary Institute, SE-751 89 Uppsala, Sweden; 4 Division of Reproduction, Department of Clinical Sciences, Swedish University of Agricultural Sciences, Box 7054, SE-750 07 Uppsala, Sweden; 5 Växa Sverige, Development and Services for Farmers, P.O. Box 2010, SE-101 24 Stockholm, Sweden

**Keywords:** *Coxiella burnetii*, Surveillance, Epidemiology, Cattle, Goat, Sheep, Moose, Cervids, Antibodies, Test evaluation

## Abstract

**Background:**

Q fever is a zoonotic disease caused by the bacterium *Coxiella burnetii*. Prevalence data in ruminant species are important to support risk assessments regarding public and animal health. The aim was to investigate the presence of or exposure to *C. burnetii* in cattle, sheep, goats and moose, and to compare two enzyme-linked immunosorbent assays (ELISAs). National surveys of antibodies against *C. burnetii* were performed for dairy cattle (n=1537), dairy goats (n=58) and sheep (n=518). Bovine samples consisted of bulk milk, caprine of pooled milk, and ovine of pooled serum. Antibodies were investigated in moose samples (n=99) from three regions. A one-year regional cattle bulk milk survey was performed on the Isle of Gotland (n=119, four occasions). Cattle, sheep and goat samples were analysed with indirect ELISA and moose samples with complement fixation test. For the sheep, goat, and parts of the cattle survey, samples were run in parallel by ELISAs based on antigens from infected ruminants and ticks. Bulk milk samples from the regional cattle survey and vaginal swabs from a subset of the sheep herds (n=80) were analysed for the agent by polymerase chain reaction. Spatial clustering was investigated in the national cattle survey.

**Results:**

The prevalence of antibodies in dairy herds was 8.2% with large regional differences. High risk clusters were identified in the southern regions. The prevalence among dairy herds on the Isle of Gotland varied from 55.9% to 64.6% and 46.4% to 58.9.0% for antibodies and agent, respectively, overall agreement between agent and antibodies was 85.2%. The prevalence of antibodies in sheep was 0.6%, the agent was not detected the vaginal swabs. Antibodies were not detected in goats or moose, although parts of the moose samples were collected in an area with high prevalence in cattle. The overall agreement between the two ELISAs was 90.4%.

**Conclusions:**

The prevalence of antibodies against *C. burnetii* in dairy cattle in Sweden shows large regional differences. The results suggest that *C. burnetii* is a rare pathogen among Swedish moose, dairy goat and sheep. ELISAs based on ruminant and tick antigen performed in a similar manner under Swedish conditions.

## Background

*Coxiella burnetii*, the cause of Q fever, is present in domestic and wild ruminants worldwide [1-6]. The presence of *C. burnetii* in domestic animal populations in Sweden is known since the early 1990’s, when the bacterium was first isolated from a sheep placenta [7]. In 1993, national abattoir surveys on Swedish sheep and cattle showed a low seroprevalence; 0.3% in sheep (n = 1001) and 1.3% in cattle (n = 784) [8]. The presence of *C. burnetii* in the Swedish goat population had not been investigated nor have studies been performed in wild ruminants.

Antibodies against *C. burnetii* are usually detected by enzyme-linked immunosorbent assays (ELISAs), indirect immunofluorescence (IFA) or by the complement fixation test (CFT). ELISAs, however, are preferred for practical reasons and for their higher sensitivity [9]. ELISAs based on antigens prepared from a ruminant isolate have been described as more sensitive than those based on antigens isolated from ticks, when assessed on goat sera [10]. Also, in scientific reports submitted to the European Food Safety Authority (EFSA) ELISAs using *C. burnetii* antigens prepared from ruminant isolates are recommended [11,12].

Prevalence data of *C. burnetii* infection in different ruminant species are important to support risk assessments or decisions on preventive measures regarding public and animal health. This study presents a series of investigations into the presence of *C. burnetii* in Swedish cattle, sheep, goats and moose. Also, a comparison of two ELISAs for the detection of antibodies against *C. burnetii* in cattle, sheep and goats is reported.

## Methods

### Study population and sampling

This study is based on five surveys done in Sweden: 1) a national survey of cattle dairy herds, 2) a regional survey of cattle dairy herds, 3) a national survey of goat dairy flocks, 4) a national survey of sheep flocks and 5) a regional survey of wild moose. Details on each of these surveys are presented below.

#### Dairy cattle herds – national survey

The prevalence of antibodies to *C. burnetii* among dairy herds was determined in a national bulk milk survey conducted in November 2008 and in June 2009. Samples were based on milk submitted for testing within the national control scheme for bovine viral diarrhoea virus, where >95% of all Swedish dairy cattle herds were included. Herds in the scheme were sampled as part of the routine milk quality testing, where samples are collected in test tubes containing Bronopol (2-bromo-2-nitropropane-1.3-diol). The samples were forwarded to the National Veterinary Institute (SVA), Uppsala, Sweden for antibody testing after the milk quality testing had been done. Every fourth milk sample was randomly selected for antibody testing. In total, samples from 1537 herds were analysed for antibodies to *C. burnetii* (2008: n = 1000; 2009: n = 537), corresponding to 26% of the Swedish dairy cattle herds (2009: n = 6020) [13]. There was no overlap in the herds selected in 2008 and 2009.

Herds that were antibody positive in 2008 (n = 85) were invited to participate in a follow up study in 2009 and 41 agreed to participate. Bulk milk from these herds was tested for antibodies and *C. burnetii* antigen. The follow up group had a similar distribution of the level of antibodies in the bulk milk when compared to the entire group of positive herds (S/P ratio =110.9 (SD 31.6) *vs* 106.7 (34.4)) and they were also geographically representative of the sampling frame. Bulk milk samples were collected directly from the tank by the farmer who was instructed to collect the sample at the end of the milking, when all lactating cows had contributed to the bulk milk.

#### Dairy cattle herds – regional survey

**Figure 1:**
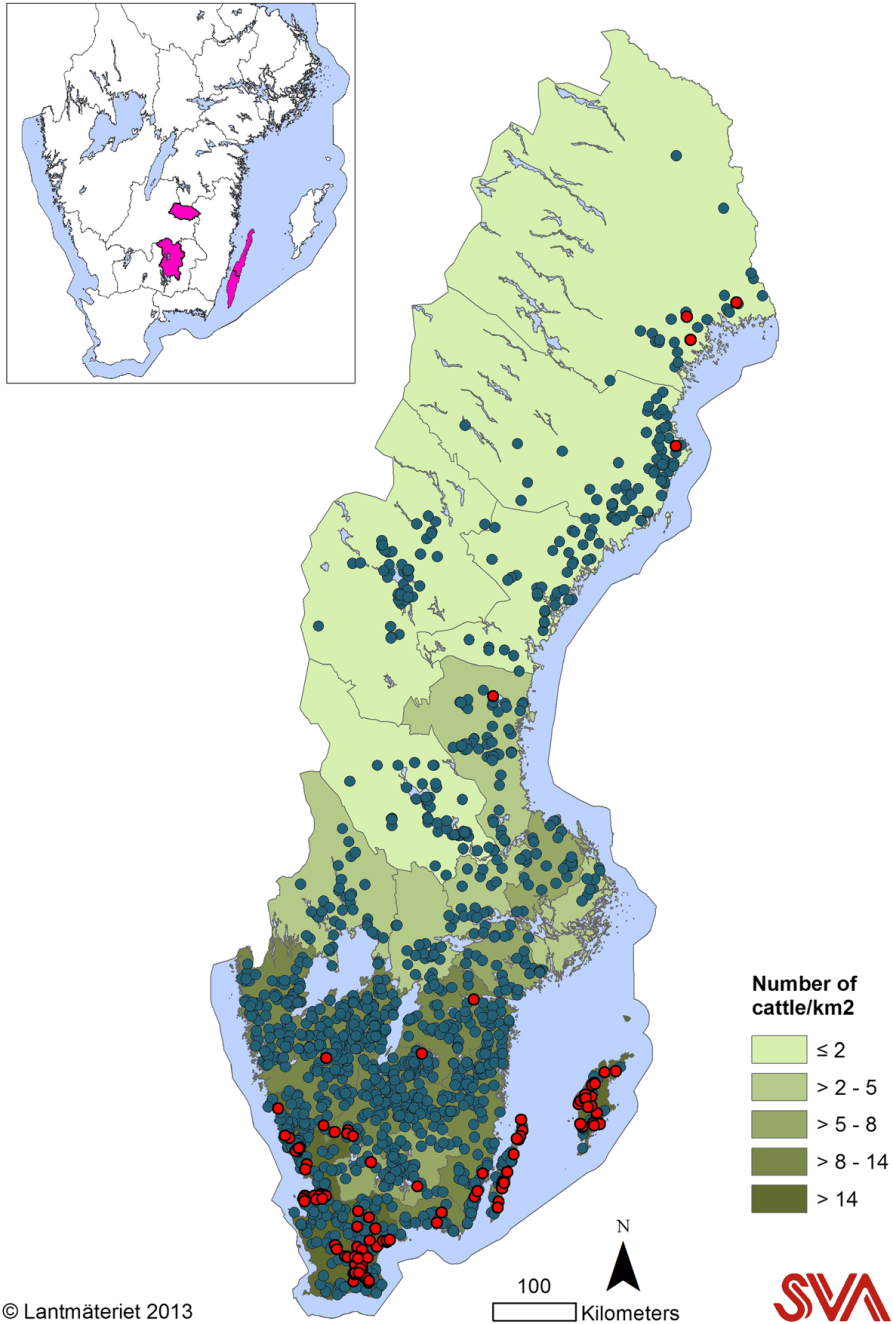
**Geographical distribution.** Dairy herd density on county level and the result of a bulk milk survey used to investigate the prevalence of antibodies against *Coxiella burnetii* in Swedish dairy cattle (2008/2009), 1411 herds tested negative (blue) and 126 positive (red). The Isle of Öland and the Isle of Gotland are the first and second island, respectively, on the right side of the mainland. The inserted figure is indicating the areas included to investigate the prevalence of antibodies to *C. burnetii* in moose.

During 2010-2011, a longitudinal, regional survey on the prevalence and incidence of antibodies as well as of the presence of *C. burnetii* DNA in bulk milk was carried out on the Isle of Gotland. This area was chosen as it was the county with the highest prevalence of antibodies in the national survey performed in 2008/2009 (Figure 1). All dairy herds on the island that were enrolled in official milk recording scheme were invited (n = 199) and all agreed to participate. The herds were sampled on four occasions in conjunction with milk quality testing; September 2010, January 2011, June 2011 and October 2011. The number of herds sampled on each occasion was 114, 119, 118 and 113, respectively. All samples were collected in test tubes containing bronopol and sent to SVA for analysis.

#### Dairy goats – national survey

The presence of antibodies and *C. burnetii* DNA in the Swedish dairy goat population was investigated during May-September 2010. Swedish goat farmers were identified through the National Sheep and Goat Register, where all holdings of goats must be registered. All farmers in the register with ≥ 10 goats (n = 205) were invited to participate. As the target population was dairy goats, farmers were also asked to state the type of goats they held and to respond irrespective of if they had dairy goats or not. In all, 92 farms held dairy goats and 58 of these agreed to participate (63%). Farmers were asked to collect milk from all lactating goats, either by sampling from the bulk tank or by manually collecting milk from each goat in a container before transfer to a sampling tube. A questionnaire was administered to collect information about herd size, the number of lactating goats and contact with sheep and cattle. The milk samples were sent to SVA, where they were analysed for antibodies and for presence of *C. burnetii* DNA.

#### Sheep – national survey

A national survey was performed in 2010 to determine the seroprevalence of antibodies to *C. burnetii* in the Swedish sheep population. The flocks were selected among flocks tested within the national maedi-visna programme; these were geographically representative of all sheep flocks affiliated to the scheme, which covers approximately 75% of the Swedish sheep population. All sheep holders must be registered in the National Sheep and Goat Register. Of a total of 819 herds sampled in the maedi-visna programme in 2010, only herds with ≥ 10 sampled animals were included in the *C. burnetii* survey (n = 510). The serum samples were pooled by herd before analysis at SVA, with 10 individuals per pool. In addition, sera were analysed individually for 54 flocks. These were purposively selectedas positive or weakly positive herds in the pooled testing (n = 3) as well as 51 negative herds, preferentially those that were borderline or from geographical areas where *C. burnetii* was known to be prevalent in cattle (based on cattle surveys in 2008-2009).

A subset of the sheep flocks included in the national survey was further investigated for *C. burnetii* DNA in 2011. Eighty herds were randomly selected from two different strata, representing different risks of having *C. burnetii* infection; 1/3 from areas where the prevalence of antibodies against *C.burnetii* was high among cattle in the 2008-2009 national bulk milk survey (Southern Sweden, incl. the Isle of Gotland) and 2/3 from other parts of Sweden. The farmers were instructed to take vaginal swabs during lambing, using Amies agar gel swabs without charcoal (Copan Venturi Transystem®, Copan Diagnostics Inc., Corona, USA). Samples were stored on the farm at around 5°C for up to 10 days until 10 sheep had lambed and were then shipped to SVA by mail. The samples were pooled by herd, before analysis for the presence of *C. burnetii* DNA.

#### Moose – regional survey

Sampling of moose was carried out as a part of a study addressing reproduction and health of moose in southern Sweden. During the first week of the moose hunting season (starting on the second Monday of October each year) in 2008-2010, post mortem blood samples were collected from shot subadult and adult moose in two areas in southern Sweden (mainland) and on the Isle of Öland (Figure 1). Dairy and sheep farms are highly abundant on the Isle of Öland, whereas the mainland moose sampling regions have a lower herd density. Hunters were advised to call field personnel immediately after the moose was shot, and blood from the heart or the caudal vena cava was collected. The blood was transported to a nearby field laboratory where it was centrifuged and stored at -20°C until analysis. Data on sex and age were recorded. Age determination of moose was performed with a previously described method [14], where the first molar was sectioned and cementum layers counted. Subadults were defined as 1.5 year old, and all moose older than 1.5 years were defined as adults.

### Laboratory analyses

After arrival at SVA, serum and milk samples were stored at -20°C and vaginal swabs at -70°C until analysis. Analyses for antibodies to *C. burnetii* in cattle, sheep and goats were performed using commercially available indirect ELISAs according to the manufacturers’ instructions. For the national and regional cattle bulk milk surveys, samples were analysed using CHEKIT Q Fever Antibody ELISA Test Kit (Idexx, Liebefeld-Bern, Switzerland), which is based on tick-derived *C. burnetii* antigen (Nine Mile strain). For the first sampling in the regional dairy cattle herd survey on the Isle of Gotland, the national goat survey, and the national sheep survey, the samples were run in parallel by CHEKIT Q Fever Antibody ELISA Test Kit and ELISA Cox kit (LSI-Laboratoire Service International, Lyon, France) based on antigen obtained from a *C. burnetii* strain isolated from sheep.

The definition of a positive sample was set according to the manufacturers’ instructions; for CHEKIT Q Fever >40 (weak positive >30 to <40), and for ELISA Cox > 40 for serum and individual milk samples and >30 for pooled milk samples. The serum samples were analysed at a dilution of 1:400 for both tests and individual and pooled milk samples were analysed by ELISA Cox at a dilution of 1:20 and by CHEKIT Q Fever at 1:5. Sensitivity of both tests is 100, and specificity 95 and 100 for ELISA Cox and CHEKIT Q Fever, respectively according to the manufacturers (Jose Hurst (LSI) and Geert Baele (Idexx), personal communications). In an investigation by Horigan et al. [15] the sensitivity was estimated to 87.0 and 98.6, and the specificity to 99.1 and 97.1 for ELISA Cox and CHEKIT Q Fever respectively. To our knowledge, there are no estimations available regarding sensitivity and specificity for pooled samples.

For detection of *C. burnetii* in the national cattle bulk milk survey, a commercial polymerase chain reaction (PCR) kit was used (Adiavet Cox PCR detection kit, Adiagene, Saint Brieuc, France). For the regional bulk milk survey of dairy herds on the Isle of Gotland, the national goat survey as well as the vaginal swab survey in sheep, an in-house real-time PCR protocol was used (Swedish Institute for Communicable Disease Control; Talar Boskani, personal communication). The two PCR protocols performed equally well when evaluated in an interlaboratory comparison of real-time PCR methods to detect *C. burnetii*[16].

Moose sera were analyzed with a complement fixation test (CFT). The CFT was based on the protocol described in the World Organization for Animal Health (OIE) Manual of Standards [9] and used antigen produced in monkey kidney tissue culture. The CFT detects both phase I and phase II antibodies, and a cut-off titre of 1:10 was used. Thus, all titres of ≤ 1:10 were considered negative in accordance with OIE standards.

### Statistical analysis

True prevalence was calculated as: (apparent prevalence + specificity - 1)/(sensitivity + specificity - 1). Concordance between ELISACox and CHEKIT Q Fever results as well as between CHEKIT Q Fever and PCR results was calculated as percent agreement and estimated by Cohen’s kappa value.

Spatial clustering of test-positive and test-negative herds in the national bulk milk survey was investigated using the spatial scan statistic (M. Kulldorff and Information Management Services, Inc. SaTScan™ version v9.1.1; http://www.satscan.org, 2011). Location of herds was based on geographical coordinates retrieved from the database of the Swedish Board of Agriculture. The testing was performed using a Bernoulli model [17] where the test-positive herds were considered cases and test-negative herds were considered controls. To be able to map a reasonable number of clusters, and clusters of reasonable extent, different input for maximum cluster size (50%, 25%, 15% and 5% of the population at risk, i.e. all sampled herds) and different cluster shapes (circular or elliptic) were attempted. In the spatial scan, the observed number of cases within each potential cluster is compared to the expected number, and the relative risk of cases within the cluster compared to outside the cluster is calculated. Whether the risk is significantly higher or lower within the cluster area compared to other areas is tested by comparing the maximum likelihood from the real dataset with maximum likelihoods from the same analysis on random replications of the data (Monte Carlo hypothesis testing, 9,999 permutations). Visualization of cluster analysis output was made using ArcMap™ version 10.0 (ESRI Inc., Redlands, CA, USA).

Statistical analyses, except for cluster analyses, were performed using the statistical software Stata (Stata statistical software: Release 12.1; College Station, TX, USA: StataCorp LP).

## Results

### Dairy cattle herds - national survey

The overall prevalence of dairy herds with antibodies to *C. burnetii* in bulk milk was 8.2% (95% confidence interval (CI) 6.9-9.7%) and the true prevalence was 7.9% based on the sensitivity and specificity determined by Horigan et al. [15]. There were large regional differences with highest prevalence on the Isles of Gotland and Öland (59% and 35%, respectively). Since the prevalence on the Isle of Gotland exceeded the highest prevalence on the continent, Gotland was selected for the regional survey. Eighty-five dairy herds were antibody positive in the 2008 bulk milk survey (8.5%) and 41 in 2009 (7.6%). The result of the national survey is shown in Figure 1.

**Figure 2:**
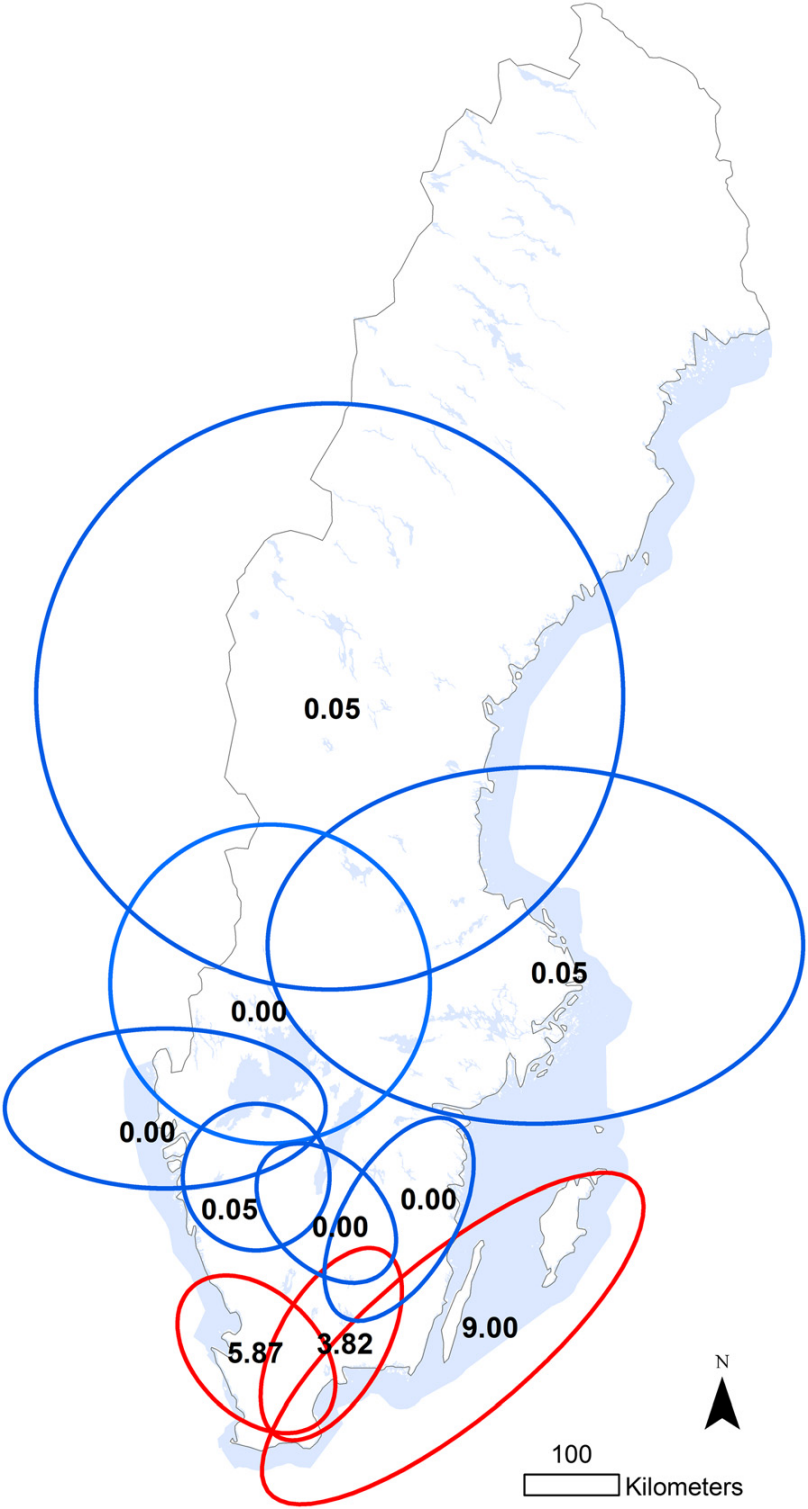
**High and low risk clusters.** Clusters with more (red lines) or less (blue lines) test positive herds than expected based on analysis of results from a bulk milk survey used to investigate the prevalence of antibodies against *Coxiella burnetii* in Swedish dairy cattle (2008/2009). Cluster analysis was based on exact coordinates and performed using the spatial scan statistic. The numbers within clusters are the calculated relative risk of each cluster compared to areas outside the cluster. ©Lantmäteriet 2013.

In the cluster analysis, several clusters with significantly higher or lower antibody prevalence were found. Applying a maximum cluster size of 15% of the population at risk was considered the most informative alternative when presenting results. Based on this, ten clusters of higher or lower prevalence than expected (*P* < 0.001) were detected. The relative risk of testing positive within clusters, compared to outside, and the location and extent of these clusters are presented in Figure 2. The relative risk of herds testing positive was 3.82 - 9.00 in the high risk clusters and in general these were located in the southern part of Sweden. Low risk clusters, on the other hand, were associated with relative risks of 0.00 - 0.046 and were located in the northern regions and in the southern inland.

Of the 85 herds that were antibody positive in 2008, 41 submitted a new bulk milk sample in 2009 for ELISA and PCR testing. The sampling was conducted 7 to 11 months after the initial test in 2008. Thirty-five herds (85%) were still antibody positive and *C. burnetii* DNA was detected in 29 of these (83%). Of the six antibody negative herds, one was PCR-positive. The PCR positive herds were all located in southern Sweden (counties of Halland, Skåne, Blekinge, Kalmar and Isle of Gotland).

### Dairy cattle herds - regional survey

**Figure 3:**
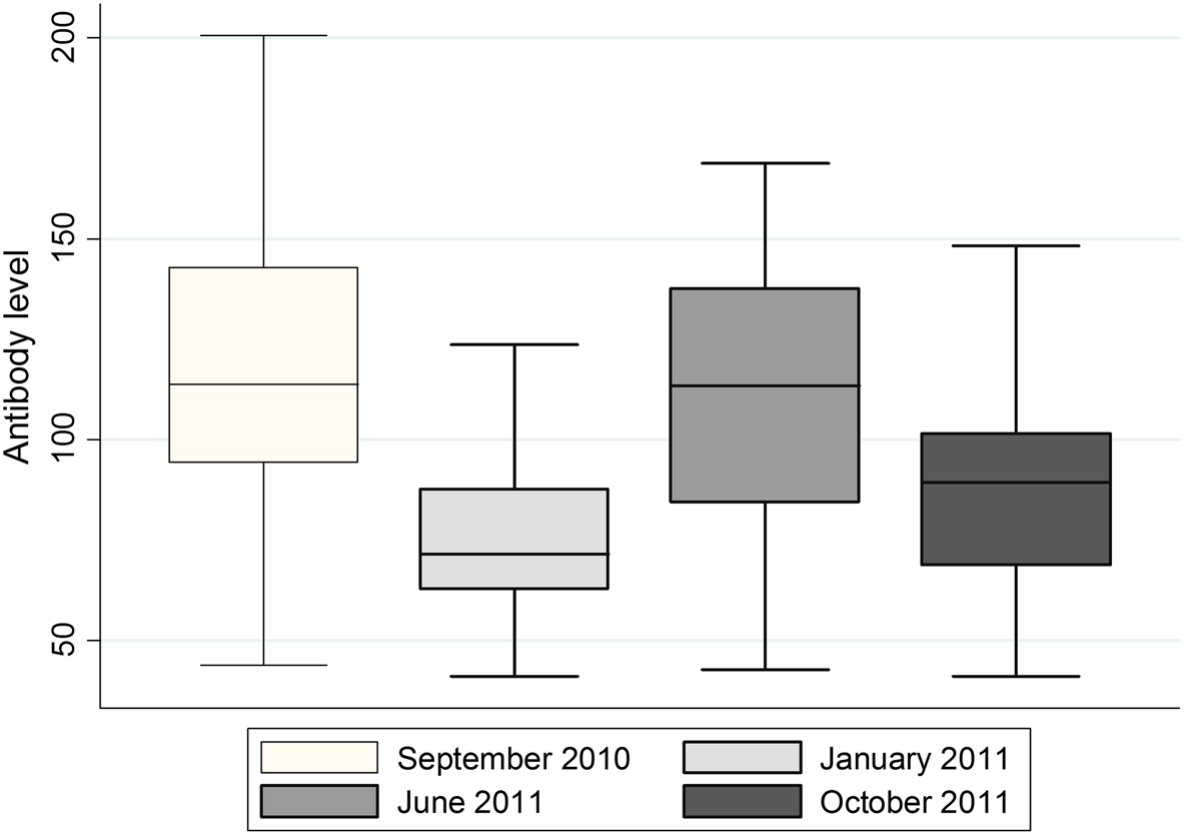
**Seasonal variation.** Levels of antibodies against *Coxiella burnetii* measured in bulk milk in 114-119 dairy herds on the Isle of Gotland, Sweden, on four sampling occasions, September 2010 to October 2011.

For the regional bulk milk survey on the Isle of Gotland, prevalence at the four sampling occasions and incidence of antibody conversion in bulk milk between these are given in Tables 1 and 2. Overall, the prevalence varied between 55.9% and 64.6% and from 46.4% to 58.9% for antibodies and *C. burnetii* DNA, respectively, and conversions from negative to positive for antibodies and/or antigen occurred in all seasons. There was a seasonal variation in antibody level in the antibody positive herds (Figure 3).

**Table 1:** Prevalence repeated sampling

	**Antibodies**			**Agent**		
	**n**	**Prevalence (%)**	**(95% CI)**	**n**	**Prevalence (%)**	**(95% CI)**
September 2010	114	61.4	(56.6 – 66.2)	113	57.8	(48.4 – 67.2)
January 2011	119	62.7	(57.8 – 67.6)	119	46.4	(47.3 – 65.5)
June 2011	118	55.9	(50.8 – 61.0)	116	55.2	(46.0 – 64.3)
October 2011	113	64.6	(55.6 – 73.6)	113	58.9	(49.7 – 68.2)

Prevalence and 95% confidence interval (CI) of antibodies against *Coxiella burnetii* and of DNA from the agent in bulk milk on four sampling occasions including 113 to 119 herds located on the Isle of Gotland, Sweden.

**Table 2:** Incidence repeated sampling

	**Antibodies**	**Agent**
September 2010 – January 2011	15.9	28.3
January 2011 – June 2011	15.9	22.0
June 2011 – October 2011	21.2	22.6

The incidence in the population at risk (negative at previous sampling) regarding detection of antibodies against *Coxiella burnetii* and bacterial DNA. Measured on four sampling occasions including 113-119 dairy herds located on the Isle of Gotland, Sweden.

In total, 453 bulk milk samples were analysed for both antibodies and *C. burnetii* DNA; 51.6% were positive for both antibody and DNA, 33.6% were consistently negative for both antibody and DNA, 9.3% were antibody positive but DNA negative and 5.5% were antibody negative but DNA positive. The overall agreement between findings of both antibodies and DNA in the bulk milk was 85.2%, with a corresponding kappa value of 0.69, indicating substantial agreement [18].

On the first sampling occasion in September 2011, the samples were run in parallel on the two different ELISAs; with a prevalence estimate of 61% (95% CI 52-70%) in the CHEKIT Q fever ELISA, and 54% (95% CI 44-63%) in the ELISACox assay. The overall agreement between the two tests was 90.4% (Table 3) and corresponding kappa value was 0.80, indicating substantial agreement [14].

**Table 3:** Antibodies parallel analysis

	**CHEKIT Q fever**		
**ELISACox, LSI**	**Positive**	**Negative**	**Total**
Positive	60	1	61
Negative	10	43	53
Total	70	44	114

Antibodies to *Coxiella burnetii* measured in bulk milk samples from 114 dairy herds, analysed with two commercial ELISAs.

### Dairy goats flocks – national survey

*C. burnetii* DNA was not detected in bulk milk from any of the 58 dairy goat flocks. Also, all investigated flocks were bulk milk antibody negative in the CHEKIT Q fever ELISA kit, whereas one herd, located in the county of Västerbotten, showed a reaction in the ELISACox kit. If the reaction would be counted as a true positive, this would correspond to an antibody prevalence of 1.7% (95% CI 0.04-9.2%) for the ELISACox test, with a true prevalence of 0.4% based on the estimations of sensitivity and specificity by Horrigan et al. [15]. The median flock size was 38 adult animals (9, 118; 5^th^, 95^th^ percentile) and the median number of lactating goats was 24 (6, 84). Contacts with sheep and cattle were reported by 16 (32%) and 19 (37%) of responders (n = 51 and 52), respectively.

### Sheep flocks – national survey

Antibodies against *C. burnetii* were detected in three of 518 sheep flocks (0.6%; 95% CI 0.1-1.7%), by one or both ELISAs; one herd was positive in both assays, one was positive only in ELISACox, and one was weakly positive only in the CHEKIT Q fever. The true prevalence calculated from the apparent prevalence of 0.4% (95% CI 0.1-1.4%; two positive in each test out of 518 herds) was 0% for both ELISAs based on the estimations of sensitivity and specificity by Horigan et al. [15]. The analysis of individual samples revealed single positive animals in the two positive pools, and two weakly positive animals in the weakly positive pool. Of the 51 negative pools, two included one positive animal, and they were both only positive in one of the two tests. Herds where antibodies were detected either in pooled analysis or among individual samples were located in the counties of Gotland, Uppsala and Jönköping. *C. burnetii* DNA was not detected in any of the 80 pooled vaginal swab samples.

### Moose – regional survey

In total, sera from 99 subadult to adult moose were collected. Mean age was 4.6 years (range 1.5-16.5, n = 45) for females and 4.6 years (range 1.5-11.5, n = 44) for males. Samples were evenly distributed based on sex and the two geographic locations (Table 4). Antibodies to *C. burnetii* were not detected in any of the samples.

**Table 4:** Sampling of moose

**Year**	**Location**	**Females**	**Males**	**Subadults (≤1.5 years)**	**Adults (>1.5 years)**
2008	Island	6	3	3	6
	Mainland	6	4	4	6
2009	Island	9	13	5	17
	Mainland	12	11	8	15
2010	Island	9	9	4	14
	Mainland	8	9	0	17
Total		50	49	24	75

Geographical, sex, and age distribution of moose sampled and analyzed for presence of antibodies against *Coxiella burnetii*.

## Discussion

Reports indicate that Sweden has a low incidence of clinical cases of Q fever in humans. In Sweden, up until 2007 only two domestic human cases had been described, in the 1980’s and 90’s [19]. In the 1990’s, a Swedish survey in humans identified 28% of sheep farmers and 13% of veterinarians to be antibody positive, indicating a larger extent of the exposure [20]. Since Q fever became a notifiable disease in humans in 2004 [21], one domestic case was reported in 2007 [19]. In 2008, eight domestic cases of Q fever in humans were reported based on serological evidence of recent exposure, all linked to a follow-up of contacts with a cattle herd with reproductive disorders [22]. The information regarding animals is sparse as very few investigations have been carried out. However, since Q fever in both humans and animals is mostly asymptomatic, its occurrence is likely to be underreported. Thirty-seven cases have been notified in cattle from 2001 to 2011 [23]. However all were reported after 2008 and mainly as a result of follow-up of the surveys reported in this paper. A targeted testing of aborted bovine fetuses carried out in 2010 did not reveal any cases positive for *C. burnetii*[23].

The prevalence of antibodies against *C. burnetii* in Swedish dairy herds varied depending on geographical region. High risk clusters were in general located in the south, which was expected since herd density is highest here. An interesting observation was that low risk clusters were identified not only in the north, were herd density is low, but also in areas in the middle parts of the Sweden were herd density is high. In a Danish survey, there was no association found between regional dairy herd density and prevalence of antibodies against *C. burnetii* in bulk tank milk [24]. Wind is known to play an important role in *C. burnetii* transmission [25] and the high risk clusters areas identified in the present study tended to be geographically located in more windy areas than the low risk clusters [26]. The longitudinal investigation on the Isle of Gotland showed that there was a high prevalence on each sampling occasion, i.e. in all seasons, both when considering detection of antibodies as well as *C. burnetii* DNA. Also, there were conversions from bulk milk negative to positive, regarding both antibodies and DNA. In the present study there was a seasonal variation in antibody titer; lower in the winter (indoor/stable season) compared to the summer (outdoor/pasture season). Grazing or contact through the fence with other ruminant herds has been shown to be associated with a higher within-herd seroprevalence [27], and the level of antibodies in bulk milk has a connection to the within-herd seroprevalence, particularly as the lowest level of antibodies was associated with the lowest mean of within-herd prevalence [28].

Swedish goats were for the first time examined for *C. burnetii* exposure. The results suggest that *C. burnetii* is a rare pathogen in this population. Swedish dairy goat flocks are largely located in the northern part of the country, where the prevalence of *C. burnetii* exposure in cattle was low as well. This may indicate similarities between the species with regard to the distribution of risk factors for transmission and persistence in this part of the country. However, it is surprising that the exposure level in sheep seems to be very low as the bacterium was indeed cultured from sheep in Sweden in the 1990’s [7]. To validate the apparent absence of exposure seen in the serological survey, the findings were followed up by screening sheep herds for vaginal *C. burnetii* excretion. The result confirmed that the prevalence of *C. burnetii* in the Swedish sheep population is likely to be very low. Still, previous studies indicate that sheep farmers have a higher prevalence of antibodies to *C. burnetii* than both veterinarians and control populations [20]. However, it is not known from those studies to what extent farmers were also exposed to cattle. Further studies on *C. burnetii* in the sheep and goat populations on the Isles of Öland and Gotland would be interesting, since the prevalence among cattle is high in these areas.

Since the cattle bulk milk survey on the Isle of Öland showed a high prevalence, we expected to find some seropositive moose in the area. This was not the case, although moose sometimes browse in the vicinity of livestock pasture. However, this mostly concerns beef cattle and not dairy cattle that generally graze closer to the farms where moose rarely are abundant. The status regarding *C. burnetii* exposure in Swedish beef cattle is still unknown. Furthermore, the density of the moose population on Isle of Öland is lower than on the mainland, i.e. two to three moose per 1,000 hectares (Magnus Johansson, personal communication) (250 – 300 moose on the whole island). On the mainland of Sweden, population density sometimes exceeds 12-14 moose per 1,000 hectares [29]. It would have been interesting to investigate the prevalence among wild ruminants on the Isle of Gotland since that was the county with the highest prevalence among dairy cattle herds; however, moose are not present there. The diagnostic method used in this study, CFT, is not validated for cervids in general and moose in particular and there is a risk of false negative results. In general, the CFT is considered to be less sensitive when compared to currently available commercial ELISAs [30], at least in the species for which it is validated. However, based on our results, we do not consider moose to be a potential reservoir or source of *C. burnetii* infection for domestic animals or humans in Sweden. Serological investigations of other cervids (including grazers) with a higher local and regional abundance, such as roe deer (with densities exceeding 100 animals per 1,000 hectares), and the development of a validated analysis could revise this conclusion.

The reason for using two parallel ELISAs in the investigations was the suggestions from the literature that assays based on antigen from *C. burnetii* isolated from ruminants rather than ticks may be more sensitive [10,12]. However, this was not found under Swedish conditions, where the two tests performed in a similar manner in sheep and goats, and where the kit with *C. burnetii* antigen of ruminant origin was, if anything, less sensitive in Swedish cattle.

In the cattle surveys, there was a consistently high correlation between presence of antibodies and detection of the agent. Although the correlation is not perfect, this indicates that bulk milk screening of antibodies can be used as a convenient tool for monitoring Q fever infection in cattle, at least under Swedish conditions.

In Sweden, veterinary and human authorities have jointly issued recommendations directed towards people in contact with herds likely or known to be infected with *C. burnetii*. These include advice on hygienic measures to reduce exposure to potentially infectious materials for both animals and people (e.g. separate calving area, cleaning and disinfection of calving area, secure disposal of placentas and aborted fetuses, etc.), and also to identify work tasks associated with a higher risk of exposure.

## Conclusions

The prevalence of antibodies against *C. burnetii* in Swedish dairy cattle shows large regional differences. The results suggest that *C. burnetii* is a rare pathogen in the Swedish moose, goat and sheep populations. ELISAs based on sheep and tick derived *C. burnetii* antigen performed in a similar manner under Swedish conditions.

## Competing interests

The authors declare that they have no competing interests.

## Authors’ contributions

AO was responsible for planning and implementation of the vaginal swab survey in sheep, for analysis of data from livestock and for drafting the manuscript. JM carried out the survey on moose and helped drafting the manuscript. JF performed the cluster analysis. GB and AA were responsible for the diagnostic work performed (serology on dairy herd follow up, regional cattle survey, sheep, goat and moose surveys; PCR analyses on cattle, sheep and goats). AMD supervised and carried out the moose survey, and AL designed and implemented all surveys apart from vaginal swab and moose, conceived the studies on livestock and helped drafting the manuscript. All authors read and approved the final manuscript.
